# Anti-angiogenic therapy or immunotherapy? A real-world study of patients with advanced non-small cell lung cancer with EGFR/HER2 exon 20 insertion mutations

**DOI:** 10.3389/fonc.2024.1357231

**Published:** 2024-03-19

**Authors:** Jiaqi Li, Mengqing Xie, Ruiying Zhao, Huiping Qiang, Qing Chang, Jialin Qian, Haijiao Lu, Yinchen Shen, Yuchen Han, Chunxia Su, Tianqing Chu

**Affiliations:** ^1^ Department of Respiratory and Critical Care Medicine, Shanghai Chest Hospital, Shanghai Jiao Tong University School of Medicine, Shanghai, China; ^2^ Department of Oncology, Shanghai Pulmonary Hospital & Thoracic Cancer Institute, Tongji University School of Medicine, Shanghai, China; ^3^ Department of Pathology, Shanghai Chest Hospital, Jiaotong University, Shanghai, China

**Keywords:** non-small cell lung cancer, anti-angiogenic, immunotherapy, rare targets, EGFR20ins, HER2-20ins

## Abstract

**Background:**

For patients with EGFR/HER2 exon20 insertions, platinum-containing double-drug chemotherapy is still the standard treatment method. First-generation TKIs have almost no therapeutic activity against EGFR exon 20 insertions. The efficacy of second-and third-generation TKIs is still controversial. Immunotherapy research is scarce, and there is an urgent need for more evidence and new treatment options for this group of patients.

**Methods:**

We reviewed patients with advanced NSCLC with EGFR/HER2 exon 20 insertion mutations treated in Shanghai Chest Hospital and Shanghai Pulmonary Hospital from 2015 to 2022 and assessed the efficacy of receiving chemotherapy, anti-angiogenic therapy and immunotherapy, including objective response rate (ORR) and disease control rate (DCR), and compared progression-free survival (PFS) and overall survival (OS).

**Results:**

Of the 126 patients included in the study, 51 patients had EGFR20ins mutations and 7 5 patients had HER2-20ins mutations. In the first-line treatment, bevacizumab + chemotherapy (Beva+Chemo), ICI+chemotherapy (ICI+Chemo), compared with chemotherapy alone (Chemo), ORR: 40% vs 33.3% vs 15% (p=0.0168); DCR: 84% vs 80.9% vs 67.5% (p=0.1817); median PFS: 8.3 vs 7.0 vs 4.6 months (p=0.0032), ICI+Chemo has a trend of benefiting on OS. Stratified analysis showed that compared with chemotherapy, ICI+Chemo was more effective for EGFR20ins mutation with median PFS: 10.3 vs. 6.3m (P=0.013); Beva+Chemo was more effective for HER2-20ins mutation, with a median PFS: 6.6 vs. 4.3m (p=0.030). In the second-line treatment of EGFR20ins mutation, bevacizumab + chemotherapy has a significant advantage in PFS compared with targeted therapy, median PFS:10.8 vs 4.0 months (P=0.016).

**Conclusion:**

For patients with EGFR20ins mutation, compared to chemotherapy, ICI+Chemo prolongs PFS, and after chemotherapy progression, bevacizumab combined with chemotherapy seems better than Furmonertinib-based targeted therapy on PFS. For HER2-20ins mutation, Beva+Chemo may be a better choice.

## 1 Introduction

The human epidermal growth factor receptor protein tyrosine kinase family, including EGFR and HER2, has emerged as an important therapeutic target for non-small cell lung cancer, breast cancer, and gastroesophageal cancer (1, 2). In NSCLC, EGFR exon 20 insertions mutations account for 1.5-2.5% of all NSCLC, 6% of EGFR-mutated NSCLC, and HER2-20 insertion mutations account for 1.5% (3–5). It is well known that EGFR exon 19 deletion and exon 21 mutation respond well to targeted therapy. However, EGFR or HER2-20 insertions are relatively difficult to treat and hardly benefits from typical EGFR TKI therapy (6–9). In the past decade, significant progress has been made in both EGFR or HER2-20 insertion fields with the development of novel TKIs, monoclonal antibodies, and antibody-drug conjugates. FDA granted accelerated approval of EGFR and MET bispecific antibody amivantamab and TKI mobocertinib as targeted drugs for EGFR exon 20 insertion in advanced non-small cell lung cancer after chemotherapy (10). Some new compounds, such as CLN-081 and DZD9008 also have shown auspicious activity, but platinum-based chemotherapy is still the primary first-line treatment for EGFR 20 insertion non-small cell lung cancer (10, 11). For HER2-20 insertion mutations, poziotinib and pyrotinib have been shown their efficacy, and the latest ADC drug, trastuzumab deruxtecan (T-DXd), has brought unprecedented tumor remission rate and control time (12). Since these TKIs or T-DXd are only used as second-line or above treatments for HER2-20 insertion patients or are just available in clinical trials, the primary first-line treatment of patients is still chemotherapy.

Our team has been paying attention to the new progress in the treatment of the EGFR mutant population and HER2-20 insertional mutant population, exploring and publishing the differences between immunotherapy and anti-angiogenesis therapy in patients with EGFR-TKI resistance (13), as well as the efficacy of immunotherapy in patients with HER2 insertional mutation (14).In this real-world retrospective cohort study, we aimed to analyze the effect of the vascular endothelial growth factor (VEGF) inhibitor bevacizumab and immune checkpoint inhibitors (ICIs) in treating patients with advanced non-small-cell lung cancer with EGFR/HER2 exon 20 insertion mutations.

## 2 Material and methods

### 2.1 Patients

The clinical data of patients with advanced NSCLC with EGFR/HER2 exon 20 insertion mutation who were treated in Shanghai Chest Hospital and Shanghai Pulmonary Hospital from 2015 to 2022 were retrospectively collected. Inclusion criteria: (1) pathologically confirmed lung adenocarcinoma; (2) EGFR/HER2 exon 20 insertion mutation detected by Amplification-Refractory MutationSystem (ARMS) method or NGS sequencing; (3) staging according to the eighth edition of TNM Systematic assessment of stage IIIb~IV; (4) at least two cycles of chemotherapy, targeted therapy, bevacizumab combination therapy or immune combination therapy in the first or second-line; (5) complete follow-up information; (6) with Evaluable image information. Exclusion criteria: (1) Other types of driver gene mutations. (2) Incomplete follow-up information or imaging records.

### 2.2 Next generation sequencing

Tissue samples of patients suffered DNA extraction and targeted sequencing, and these tests were performed in Burning Rock Biotech, a commercial clinical laboratory accredited by the College of American Pathologist (CAP) and certified by the Clinical Laboratory Improvement Amendments (CLIA). The tests according to the instructions, in detail, DNA of tissue samples were extracted by QIAamp DNA Kit(Qiagen, 51306), and peripheral white blood cells (WBCs) were separated by centrifugation at 1,800×g 10 min at 4°C within 2 h after blood collection, and WBCs were extracted genomic DNA as the germline controls.

DNA fragmentation was performed by Covaris M220, then suffered end repair, phosphorylation, and adaptor ligation. DNA fragments within 200–400 bp size were selected by a magnetic bead (Agencourt AMPure XP Kit, Beckman Coulter, California, USA), then followed by hybridization with capture probes baits, hybrid selection with magnetic beads, and PCR amplification. Then the quality and size of the fragments were evaluated by a high-sensitivity DNA assay (Bioanalyzer 2100, Agilent Technologies, CA, USA). Ultimately, indexed samples were sequenced on Nextseq500 sequencer (Illumina, Inc., California, USA) with pair-end reads and an average sequencing depth of 1,000×. Genomic profiling was performed using a panel covering 68 lung cancer-related genes (Burning Rock Biotech Ltd.).

### 2.3 Sequence data analysis

The sequence data were mapped to the reference human genome (hg19) by Burrows-Wheeler Aligner version 0.7.10. Local alignment optimization, duplication marking, and variant calling were performed by Genome Analysis Tool Kit version 3.2 and VarScan version 2.4.3. Tissue samples were compared against their own WBCs control to identify somatic variants. Variants with population frequency over 0.1% in the ExAC, 1000 Genomes, dbSNP, or ESP6500SI-V2 databases were grouped as single nucleotide polymorphisms (SNPs) and excluded from further analysis. The remaining variants were annotated with ANNOVAR (2016-02-01 release) and SnpEff version 3.6. DNA translocation analysis was performed using both Tophat2 and Factera 1.4.3.

### 2.4 ARMS-PCR

Suction of DNA from formalin fixed paraffin embedded (FFPE) with TRIzol ^®^reagent (cat. no. 15596–026; Invitrogen; Thermo Fisher Scientific, Inc.), according to the manufacturer’s protocols. Determination of DNA concentration of all samples at 280nm using NanoDropND-1000 spectrophotometer (Thermo Fisher Scientific, Inc.). The gene mutations in these samples were detected by amplification refractory mutation system-polymerase chain reaction (ARMS-PCR) and the thermo-cycling conditions of PCR were as follows: 1 cycle of 95°C for 5 min; followed by 15 cycles of 95°C for 25 s, 64°C for 20 s, 72°C for 20 s; and then finally 31 cycles of 93°C for 25 s, 60°C for 35 s, 72°C for 20 s. The ARMS-PCR reagents were provided by Burning Rock Biotech Ltd.

### 2.5 Efficacy assessment

Objective response rate (ORR) and disease control rate (DCR) were assessed according to Response Evaluation Criteria in Solid Tumors (RECIST) version 1.1 Progression-free survival period (PFS)was defined as the time from initiation of treatment to disease progression or death, and overall survival (OS) was defined as the time from initiation of treatment to death from any cause. The data cutoff was October 2022, and patients with an ongoing response at this time or the last date of follow-up were considered censored.

### 2.6 Statistical analysis

Two groups of continuous variables were analyzed by Student’s t-test, and multiple groups were analyzed by Analysis Of Variable; categorical variables were analyzed by Fisher’s or Pearson’s χ2 test. Median PFS and OS were assessed by the Kaplan-Meier method, and the log-rank test was used to assess the difference in survival between the two groups. All tests were two-sided, and P values < 0.05 were considered statistically significant. Statistical analysis was performed using SAS (version 3.1) and R (version 4.0.4).

## 3 Results

### 3.1 Patient characteristics

A total of 126 patients, including 51 EGFR exon 20 insertion mutation patients and 75 HER2 exon 20 insertion mutation patients from Shanghai Chest Hospital and Shanghai Pulmonary Hospital were enrolled. The median age of all patients was 61 years (range 30 to 83), of whom 52.5% were female, and 77.8% were never smokers.

For the first-line treatment, 80 patients received chemotherapy alone(Chemo)(Pemetrexed+Carboplatin: 56, Pemetrexed+Cisplatin: 17, Paclitaxel+Carboplatin: 1, Gemcitabine+Cisplatin: 2, Paclitaxel+Cisplatin: 4), 25 patients received bevacizumab + chemotherapy(Beva+Chemo), and 21 patients received ICI+ chemotherapy(ICI+Chemo)(Nivolumab: 4, Pembrolizumab: 12, Atezolizumab: 3,Camrelizumab: 2); in the second-line treatment, 17 patients received bevacizumab+chemotherapy, 27 patients received targeted therapy(Poziotinib:1, Furmonertinib:6, Afatinib:8, Pyrotinib:10, Gefitinib:1, Mobocertinib:1), and 28 received ICI monotherapy or combination therapy(Sintilimab:5, Nivolumab:9, Camrelizumab:6, Pembrolizumab:7, Terlizumab:1), the remaining patients did not receive second-line treatment or were lost to follow-up. The clinical characteristics between the groups remained balanced, as detailed in **Table 1**.

**Table 1:** Baseline demographic and clinical characteristics of patients stratified by treatment strategies.

Characteristics	Total	First Line(n=126)			P	Second Line(n=72)			P value
	(n=126)	Chemo (n=80)	Beva+Chemo	ICI+Chemo	value	Target	Beva+Chemo	ICI	
			(n=25)	(n=21)		(n=27)	(n=17)	(n=28)	
Age,years,median[range]	61[30-83]	61[35-83]	61[30-79]	58[35-81]		61[36-83]	57[45-73]	56[39-69]	
Gender,n(%)									
Male	51(40.5)	39(49.0)	11(44.0)	10(47.6)	0.9180	14(51.9)	5(29.4)	15(53.6)	0.2455
Female	75(59.5)	41(51.0)	14(56.0)	11(52.4)		13(48.1)	12(70.6)	13(46.4)	
Smoking Status,n(%)									
Current/fomer	26(20.6)	22(27.5)	2(8.0)	2(9.5)	0.0435	7(25.9)	2(11.8)	9(32.1)	0.3120
Never	100(79.4	58(72.5)	23(92.0)	19(90.5)		20(74.1)	15(88.2)	19(67.9)	
Mutation types,n(%)					0.1722				0.4375
EGFR20ins	51(40.5)	28(35.0)	14(56.0)	9(42.9)		9(33.3)	9(53.0)	12(42.9)	
Her2-20ins	75(59.5)	52(65.0)	11(44.0)	12(51.7)		18(66.7)	8(47.1)	16(57.1)	
PD-L1 status,n(%)									
≥1%	16(12.7)	8(10.0)	4(16.0)	4(19.0)	0.5699	4(14.8)	0(0)	7(25.0)	0.1310
<1%	38(30.2)	26(32.5)	5(20.0)	7(33.3)		6(22.2)	6(35.3)	10(35.7)	
Not examined	72(57.1)	46(57.5)	16(64.0)	10(47.6)		17(63.0)	11(64.7)	11(39.3)	
No.of metastatic sites,n(%)									
0-1	59(46.8)	40(50.0)	14(56.0)	5(23.8)	0.0934	11(40.7)	8(47.1)	12(42.9)	0.7282
2	37(29.4)	25(31.3)	5(20.0)	7(33.3)		8(29.6)	6(35.3)	6(21.4)	
≥3	30(23.8)	15(18.7)	6(24.0)	9(42.9)		8(29.6)	3(17.6)	10(35.7)	
ECOG PS									
0-1	120(95.2)	74(92.5)	25(100.0)	21(100)	0.1658	26(96.3)	16(94.1)	27(100)	0.4908
≥2	6(4.8)	6(7.5)	0(0)	0(0)		1(3.7)	1(5.9)	0(0)	

### 3.2 Efficacy of treatment strategies in first-line therapy

**Figure 1:**
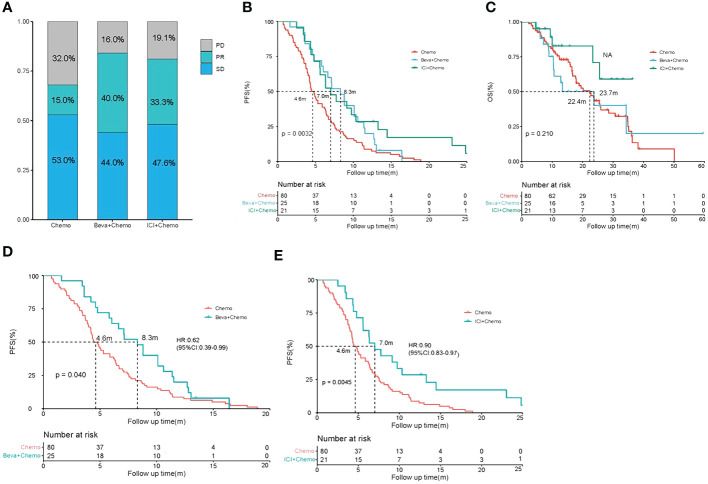
Best overall response (BOR), progression-free survival (PFS), and overall survival (OS) comparison of patients. **(A–C)** BOR, PFS, and OS of patients who received chemotherapy(Chemo),chemo-bevacizumab combination(Beva+Chemo), and chemo-immunotherapy combinations(ICI+Chemo); **(D)** PFS of patients who received chemotherapy(Chemo) and chemo-bevacizumab combination(Beva+Chemo); **(E)** PFS of patients who received chemotherapy(Chemo) and chemo-immunotherapy combinations(ICI+Chemo).

Among the first-line treatments for all enrolled patients, 80 received chemotherapy alone, 25 received Beva+Chemo, and 21 received ICI+Chemo. Beva + Chemo vs. Chemo, ORR: 40% vs. 15% (P=0.0073), DCR: 84% vs. 67.5% (p=0.1109), median PFS: 8.3 vs. 4.6m [HR: 0.62 (95%CI: 0.39-0.99), p=0.040]; median OS: 23.7 vs. 22.4m (p=0.750) (**Figures 1A–C**). ICI+Chemo vs. Chemo, ORR: 33.3% vs. 15% (p=0.0667), DCR: 80.9% vs. 68% (p=0.2299), median PFS: 7.0 vs. 4.6m, [HR: 0.90 (95%CI: 0.83-0.97), p=0.0045]; OS has a trend of benefiting compared with chemotherapy. ICI+Chemo vs. Beva+Chemo, ORR: 33.3% vs. 40.0% (p=0.6408), DCR:80.9% vs. 84%(p=0.7859), median PFS: 8.3 vs. 7.0m (P=0.440) (**Figures 1D, E**). Compared with chemotherapy alone, both Beva+Chemo and ICI+Chemo could prolong PFS, while Beva+Chemo had no difference in PFS compared with ICI+Chemo.

**Figure 2:**
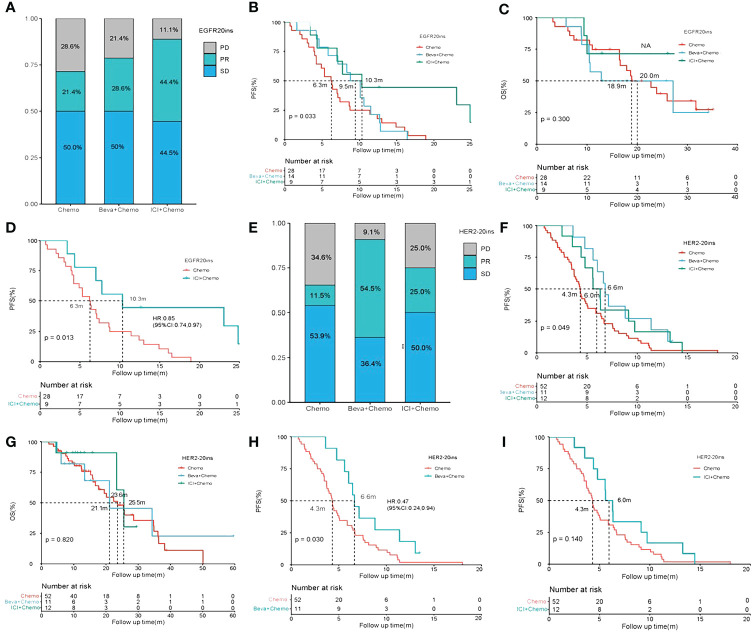
Results of stratified analysis efficacy of various treatment regimens in EGFR20ins and HER2-20ins. **(A–C)** BOR, PFS, and OS comparison of chemotherapy (Chemo),chemo-bevacizumab combination (Beva+Chemo) and chemo-immunotherapy combinations(ICI+Chemo) in patients with EGFR20ins; **(D)** PFS comparison of chemotherapy (Chemo) and chemo-immunotherapy combinations(ICI+Chemo) in patients with EGFR20ins; **(E–G)** BOR, PFS and OS comparison of chemotherapy (Chemo),chemo-bevacizumab combination (Beva+Chemo) and chemo-immunotherapy combinations(ICI+Chemo) in patients with HER2-20ins; **(H)** PFS comparison of chemotherapy (Chemo) and chemo-bevacizumab combination (Beva+Chemo) in patients with HER2-20ins. **(I)** PFS comparison of chemotherapy(Chemo) and chemo-immunotherapy combinations(ICI+Chemo) in patients with HER2-20ins.

The results of the stratified analysis showed that among the first-line treatments for all enrolled patients, 51 patients had EGFR20ins mutation, of which 28 received chemotherapy alone, 14 received Beva + Chemo, and 9 received ICI+Chemo. Compared to all three, ORR: 21.4% vs. 28.6% vs. 44.4% (p=0.4090); DCR: 71.4% vs. 78.6% vs. 88.9% (p=0.5549) (**Figure 2A**). Beva+ Chemo vs.Chemo, median PFS: 9.5 vs. 6.3 (P=0.600), median OS: 20.0 vs. 18.9m (P=0.600). Compared with chemotherapy, ICI+Chemo has a longer median PFS: 10.3 vs. 6.3m (P=0.013). Beva+Chemo compared with ICI+Chemo, median PFS: 9.5 vs. 10.3m (P=0.110), there was no significant difference in OS (**Figures 2B–D**).

Among the 75 patients with HER2-20ins mutation, 52 were treated with chemotherapy alone, 11 with Beva + Chemo, and 12 with ICI + Chemo. ORR: 11.5% vs. 54.5% vs. 25.0% (p=0.0051). The difference in ORR was statistically significant for Beva + Chemo compared to chemotherapy: 54.5% vs. 11.5% (p= 0.001) and not statistically significant for ICI + Chemo 54.5% vs 25.0%. (p=0.1470). DCR: 65.4% vs. 90.9% vs. 75% (p=0.2296) (**Figure 2E**). Compared to the three, the median PFS:4.3 vs. 6.6 vs. 6.0m (p=0.049) and median OS:23.6 vs. 21.1 vs. 25.5m (p=0.820) (**Figures 2F, G**). Beva+Chemo have a benefit compared to Chemo, with a median PFS of 6.63 vs. 4.31m (p=0.030). Compared to chemotherapy, ICI + Chemo did not appear to have an advantage in terms of PFS (p=0.140) but won the longest median OS, although there was no statistical difference (**Figures 2G–I**).

In addition, the two mutation types of EGFR20ins and HER2-20ins have different responses to the same treatment regimen. Among the 80 patients who received first-line chemotherapy, 28 patients had EGFR20ins, compared with HER2-20ins (52 patients), median PFS: 6.3 vs. 4.3 (P=0.026), median OS: 18.9 vs. 23.6 (P=0.670), and compared with the HER2- 20ins mutation, the EGFR20ins was more sensitive to chemotherapy, and the PFS benefited significantly, although there was no statistical difference in OS (**Supplementary Figures 1A, B**). Among the 25 patients who received beva+chemo, EGFR20ins (14 patients) compared with HER2-20ins (11 patients), median PFS: 9.5 vs. 6.6m (P=0.700); median OS: 11.7 vs. 21.1m (P=0.410), both No statistical difference (**Supplementary Figures 1C, D**). Compared with EGFR20ins and HER2-20ins receiving ICI+ chemotherapy, median PFS: 10.3 vs. 6.0m (P=0.140) (**Supplementary Figures 1E, F**).

### 3.3 Efficacy of treatment strategies in second-line therapy

**Figure 3:**
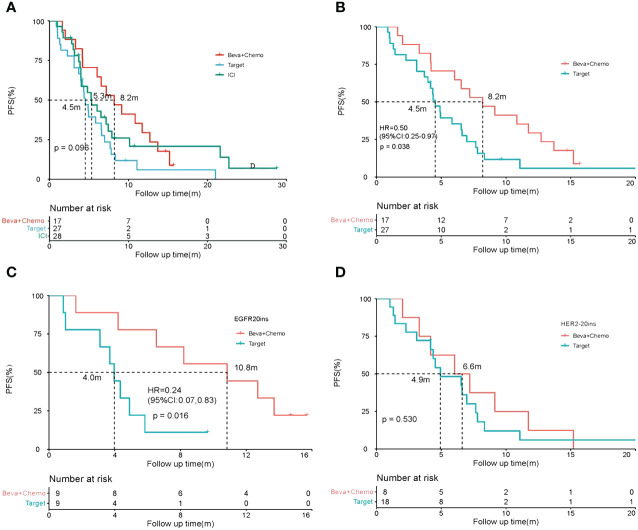
Efficacy of treatment strategies in second-line therapy. **(A)** PFS comparison of Beva+Chemo, targeted therapy (Target), and immunotherapy(ICI) in the whole cohort; **(B)** PFS comparison of Beva+Chemo and targeted therapy (Target) in the whole cohort; **(C, D)** Results of stratified analysis: PFS of Beva+Chemo and targeted therapy(Target) in patients with EGFR20ins and HER2-20ins.

In the second-line treatment, 17 patients received beva+chemo, 27 received targeted therapy, and 28 received ICI monotherapy or combination therapy. The median PFS of the three groups was 8.2 vs. 4.5 vs 5.3m(p=0.096) (**Figure 3A**). Compared with targeted therapy, beva+chemo had a significantly longer median PFS (8.2 vs. 4.5m, HR=0.50, P=0.038). The results of the stratified analysis showed that among the 18 patients with EGFR20ins, a significantly longer PFS was observed in the beva+chemo group (9 patients) compared with the targeted therapy (9 patients) group (10.8 vs. 4.0m, P=0.016). This phenomenon was not seen in the HER2-20 insertion cohort. (**Figures 3B–D**).

**Figure 4:**
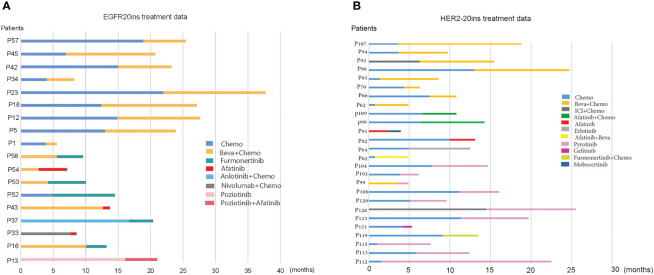
First- and second-line treatment strategies for patients with EGFR20ins and HER2-20ins. **(A)** First- and second-line treatment strategies for patients with EGFR20ins; **(B)** First- and second-line treatment strategies for patients with HER2-20ins.

We then analyzed the first- and second-line treatment regimens of these 44 patients who received beva+chemo (17 patients) or targeted therapy (27 patients) in the second line, as shown in **Figure 4**. Among the 18 patients with EGFR20ins, the median PFS of 9 patients treated with Beva+Chemo as second-line treatment was 10.8 months; among various second-line targeted therapy strategies, Furmonertinib had the best effect, with a median PFS of 4.0 months (5 patients). Afatinib had little effect in two patients (**Figure 4A**). Among the 26 patients with HER2-20ins, 8 received Beva+Chemo, 3 received Target+Chemo, and 10 received Pyrotinib monotherapy. Among them, the median PFS of patients treated with Beva + Chemo was 6.6 months, the median PFS of Pyrotinib alone was also 6.6 months, and the efficacy of the two was comparable. (**Figure 4B**)(The targeted drugs involved that have not yet been approved in China are purchased and taken by patients themselves or participate in clinical trials.)

### 3.4 Efficacy of treatment strategies in patients with specific metastatic sites

Of the patients treated in the first line, 45 had bone metastases, 21 had brain metastases, and 12 had liver metastases. For patients with bone metastases, Beva + Chemo compared with chemotherapy alone and ICI + chemotherapy, median PFSs were 7.1 vs. 4.3 vs. 6.2m (P=0.490). Among patients with brain metastases, the median PFS compared among the three groups was 8.8 vs. 5.1 vs. 5.5m (p=0.690). For patients with liver metastases, ICI+chemotherapy had the most prolonged median PFS among the three regimens [ICI+chemo vs. Chemo vs. Beva+chemo: 7.0 vs. 6.4 vs. 5.4m (P=0.490)]. However, these differences were not statistically significant. (**Supplementary Figure 2**).

### 3.5 Impact of EGFR 20 exon insertion location on the efficacy of treatment strategies

We defined the 762-764 amino acid region as the front-end insertion, and the insertion after 767 is called the back-end insertion (12).In the first-line chemotherapy group, one patient carrying EGFR20 exon front-end insertion had a median PFS of 6.4m, and one patient in the ICI+chemo group had a median PFS of 9.6m. In the Beva+chemo group, three patients with EGFR20 exon front-end insertion and 11 with EGFR20 exon back-end insertion. The median PFS was not significantly different: 8.8 vs. 10.6m (P=0.490) (**Supplementary Figure 3A**).The specific types of exon 20 insertion mutations are provided in **Supplementary Table 1 of the Supplementary Materials**.

### 3.6 The effect of PD-L1 expression status on the efficacy of immunotherapy.

Among all 48 patients who received first- or second-line immunotherapy, 14 had negative PD-L1 expression, and 16 had the positive expression, compared with median PFS: 7.2 vs. 7.6m (p=0.380). Patients with positive PD-L1 expression have a more pronounced trend of benefit. (**Supplementary Figure 3B**).

## 4 Discussion

This retrospective study included 126 advanced lung adenocarcinoma patients with EGFR/HER2 exon 20 insertion mutations, of which EGFR20ins accounted for 40.5%, and HER2-20ins accounted for 59.5%; the proportion of women and never-smokers was higher, were 59.5% and 79.4%, respectively, which is consistent with the previous report (10, 15). In the first-line treatment, the vast majority of patients received chemotherapy, accounting for 63.5%, 25 patients (19.8%) received Beva+Chemo, and 21 patients (16.7%) received ICI+Chemo. In the entire cohort, both Beva + Chemo and ICI + Chemo prolonged PFS compared with chemotherapy alone, and there was no difference between Beva+ Chemo and ICI + Chemo; the results of the stratified analysis showed that in EGFR20ins, compared with chemotherapy, ICI+Chemo can prolong PFS while Beva+Chemo has no apparent benefit. In HER2-20ins, on the contrary, Beva+ Chemo had a benefit in PFS compared with chemotherapy, while ICI+ Chemo had no difference compared with chemotherapy. As for ICI+ chemotherapy, patients with EGFR20ins mutations can achieve longer PFS, while the limited efficacy in HER2-20ins may be due to differences in the tumor microenvironment of the two mutations. The analysis results of chemotherapy also showed that the response of EGFR20ins to chemotherapy was better than that of HER2-20ins, which may reflect the difference in the tumor microenvironment of these two mutation types on the other hand. After analyzing the immune microenvironment of EGFR 20 exon mutations and HER2-20 exon mutations, researchers found differences in the number of cytotoxic cells, Th1 cells, and NK cells lacking CD56dim. In addition, the co-stimulatory molecules expressed in the tumor samples with the two mutations were also different (16).Combined with our study, we speculate that the tumor microenvironment of EGFR20ins may be more suitable for immunotherapy and chemotherapy, while the tumor microenvironment of HER2-20ins perhaps benefit more from anti-vascular therapy, which requires more clinical data and basic research to verify.

For patients with EGFR20ins, after progression on first-line chemotherapy, the median PFS of Beva+ Chemo was 10.8 months, comparable to the median PFS (9.5months) of first-line Beva+ Chemo and better than Furmonertinib-based targeted therapy (median PFS: 10.8 vs. 4.0m, P=0.016). Among the various targeted therapy strategies, Furmonertinib was the most effective, with a median PFS of 4.0 months (5 patients). Three patients received afatinib, two of whom had no response (genotypes p.P772_H773delinsHNPY and p.A767_V769dup, respectively). A previous study reported that mutation types other than the p.A763_Y764insFQEA site mutation were almost unresponsive to afatinib, and our results are consistent with previous studies (17). A patient who received bevacizumab combined with afatinib in the first-line treatment had a PFS of 2.8 months. After the first-line treatment progressed, the patient refused the third-generation TKI and continued to take afatinib. The second-line PFS reached 4.3 months (genotype p.A767_V769dup). Whether bevacizumab increases the sensitivity of afatinib treatment has not been previously reported, and more data are needed to verify. We also found that a patient numbered 1 had a poor response to Beva+ Chemo, the patient’s genotype was p.Asn771_His773dup, and further analysis of the EGFR20 exon insertion site found that the front-end insertion was compared with the back-end insertion. There was no significant difference compared to PFS (median PFS: 10.6 vs. 8.8m, p=0.490). For patients with HER2-20ins, second-line Beva+Chemo was comparable to Pyrotinib-based targeted therapy (median PFS: 6.6 vs. 4.9m, p=0.530).

In conclusion, our study found that for patients with advanced lung adenocarcinoma with HER2 exon 20 insertion mutation, bevacizumab combined with chemotherapy can prolong PFS compared with chemotherapy alone. For patients with EGFR20ins mutation, ICI+Chemo looks like the better option. After chemotherapy progression, bevacizumab combined with chemotherapy appears better than Furmonertinib-based targeted therapy in PFS. Compared with HER2-20ins mutation, patients with EGFR20ins mutation look more sensitive to chemotherapy. The interpretation of our findings must consider that this is a retrospective study, and the small sample size in the subgroup analysis may result in biased conclusions. Second, some patients were included in both first-line and second-line analyses. Second-line efficacy may be affected by the synergistic effect of delayed first-line treatment for these overlapping patients. In addition, as a retrospective study, patients lost to follow-up are unavoidable but influencing factors that need to be considered.

## Data availability statement

The original contributions presented in the study are included in the article/**Supplementary Material**. Further inquiries can be directed to the corresponding authors.

## Ethics statement

This study was approved by the Ethics Committees of Shanghai Chest Hospital. The studies were conducted in accordance with the local legislation and institutional requirements. The participants provided their written informed consent to participate in this study.

## Author contributions

JL: Methodology, Formal analysis, Data curation, Writing – review & editing, Writing – original draft. MX: Writing – review & editing, Writing – original draft, Data curation. RZ: Writing – review & editing, Writing – original draft, Data curation. HQ: Writing – review & editing, Data curation. QC: Writing – review & editing, Data curation. JQ: Writing – review & editing, Data curation. HL: Writing – original draft, Data curation. YS: Writing – review & editing, Supervision. YH: Writing – original draft, Supervision. CS: Writing – review & editing, Supervision. TC: Writing – original draft, Writing – review & editing.

## Supplementary material

Supplementary Figure 1 Comparison of the efficacy of EGFR20ins and HER2-20ins on the same treatment regimen. **(A, B)** Comparison of PFS and OS in EGFR20ins and HER2-20ins patients receiving chemotherapy; **(C, D)** Comparison of PFS and OS in EGFR20ins and HER2-20ins patients receiving Beva+Chemo. **(E, F)** Comparison of PFS and OS in EGFR20ins and HER2-20ins patients receiving ICI+Chemo.

Supplementary Figure 2 Comparison of various treatment strategies for patients with specific metastatic sites. **(A)** PFS of three treatment strategies in patients with bone metastases; **(B)** PFS of three treatment strategies in patients with brain metastases; **(C)** PFS of three treatment strategies in patients with liver metastases.

Supplementary Figure 3 Comparison of efficacy in different populations. **(A)** Comparison of PFS in patients with EGFR 20 exon front-end insertion and back-end insertion receiving Beva+Chemo. **(B)** Comparison of PFS between PD-L1 negative and positive patients receiving immunotherapy.
