# Supplementary material for: Anti-angiogenic therapy or immunotherapy? A real-world study of patients with advanced non-small cell lung cancer with EGFR/HER2 exon 20 insertion mutations
Source: Front Oncol. 2024 Mar 19;14:1357231. doi: 10.3389/fonc.2024.1357231 (PMC10985835; doi:10.3389/fonc.2024.1357231)
Supplement: Supplementary file 1 [file Table_1.docx]

Supplementary materials

Table S1 Table of exon 20 insertion types

|  | Patient's  ID | Types of exon20 insertion |
| --- | --- | --- |
| EGFR20ins | 1 | NM_005228.c.2311_2319dupAACCCCCACp.Asn771_His773dup |
|  | 2 | NM_005228.3 c.2314_2319dupCCCCAC p.Pro772_His773dup |
|  | 3 | NM_005228.3 c.2300_2308dupCCAGCGTGG p.Ala767_Val769dup |
|  | 4 | NM_005228.3 c.2313delinsAGGT p.Asn771delinsLysGly |
|  | 5 | NM_005228.3 c.2310_2311insGGGTTA p.Asp770_Asn771insGlyLeu |
|  | 6 | NM_005228.3 c.2303_2311dup p.Ser768_Asp770dup |
|  | 7 | NM_005228.3 c.2310_2311insGGG p.Asp770_Asn771insGly |
|  | 8 | NM_005228.3 c.2308_2309insGTTGTGTGG p.Val769_Asp770insGlyCysVal |
|  | 10 | NM_005228.3，c.2303_2311dup，p.Ser768_Asp770dup |
|  | 11 | NM_005228.3 c.2300_2308dup p.Ala767_Val769dup |
|  | 12 | NM_005228.3,c.2300_2308dup，p.Ala767_Val769dup |
|  | 13 | NM_005228.3,c.2303_2311dup(p.Ser768_Asp770dup) |
|  | 15 | NM_005228.3 c.2284-5_2290dup p.Ala763_Tyr764insPheGlnGluAla |
|  | 16 | NM_005228.3 c.2308_2309insGCGGCACAC p.Asp770delinsGlyGlyThrHis |
|  | 17 | NM_005228.3 c.2310_2311insGGT(p.Asp770_Asn771insGly) |
|  | 18 | NM_005228.3 c.2303_2311dup p.Ser768_Asp770dup |
|  | 19 | NM_005228.3 c.2300_2308dup p.Ala767_Val769dup |
|  | 20 | NM_005228.3，c.2284-5_2290dup，p.Ala763_Tyr764insPheGlnGluAla |
|  | 22 | NM_005228.3，c.2311_2319dup，p.Asn771_His773dup |
|  | 23 | NM_005228.3,c.2315_2320dup(p.His773_Val774insAlaHis) |
|  | 24 | NM_005228.3,c.2300_2308dup(p.Ala767_Val769dup) |
|  | 25 | NM_005228.3, c.2308_2309insGTT(p.D770delinsGY) |
|  | 26 | NM_005228.3, c.2300_2308dup(p.A767_V769dup) |
|  | 27 | NM_005228.3, c.2317_2319dup(p.H773dup) |
|  | 28 | NM_005228.3, c.2290_2291insTCCGGGAAGCCT(p.A763_Y764insFREA) |
|  | 29 | NM_005228.3, c.2302_2303insCGCTGGCCA(p.A767_S768insTLA) |
|  | 31 | NM_005228.3, c.2284-5_2290dup(p.A763_Y764insFQEA) |
|  | 32 | NM_005228.3, c.2314_2319dup(p.P772_H773dup) |
|  | 33 | NM_005228.3, c.2300_2308dup(p.A767_V769dup) |
|  | 34 | NM_005228.3, c.2310_2311insGGT(p.D770_N771insG) |
|  | 35 | NM_005228.3, c.2315_2320dup(p.H773_V774insAH) |
|  | 36 | NM_005228.3, c.2303_2311dup(p.S768_D770dup) |
|  | 37 | NM_005228.3, c.2300_2308dup(p.A767_V769dup) |
|  | 39 | NM_005228.3, c.2303_2311dup(p.S768_D770dup) |
|  | 41 | NM_005228.3, c.2311_2319dup(p.N771_H773dup) |
|  | 42 | NM_005228.3, c.2284-5_2290dup(p.A763_Y764insFQEA) |
|  | 43 | NM_005228.3, c.2315_2317delinsACAACCCCT(p.P772_H773delinsHNPY) |
|  | 44 | NM_005228.3, c.2314_2319dup(p.P772_H773dup) |
|  | 45 | NM_005228.3, c.2300_2308dup(p.A767_V769dup) |
|  | 46 | NM_005228.3, c.2317_2319dup(p.H773dup) |
|  | 47 | NM_005228.3, c.2284-5_2290dup(p.A763_Y764insFQEA) |
|  | 48 | NM_005228.3, c.2300_2308dup(p.A767_V769dup) |
|  | 49 | NM_005228.3, c.2300_2308dup(p.A767_V769dup) |
|  | 50 | NM_005228.3, c.2300_2308dup(p.A767_V769dup) |
|  | 51 | NM_005228.3, c.2303_2311dup (p.S768_D770dup) |
|  | 52 | NM_005228.3, c.2300_2308dup(p.A767_V769dup) |
|  | 53 | NM_005228.3, c.2284-5_2290dup(p.A763_Y764insFQEA) |
|  | 54 | NM_005228.3, c.2300_2308dup(p.A767_V769dup) |
|  | 56 | NM_005228.3, c.2300_2308dup (p.A767_V769dup) |
|  | 57 | NM_005228.3, c.2317_2319dup (p.H773dup) |
|  | 58 | NM_005228.3, c.2310_2311insGGG (p.D770_N771insG) |
| HER2 20ins | 61 | NM_004448.c.2313_2324dupATACGTGATG（p.Ala775_Gly776insTyrValMetAla |
|  | 62 | NM_004448.3 c.2326_2327insTGTp.Gly776delinsValCys |
|  | 63 | NM_004448.3 c.2313_2324dupATACGTGATGGC p.Ala775_Gly776insTyrValMetAla |
|  | 64 | NM_004448.3，c.2313_2324dup，p.Ala775_Gly776insTyrValMetAla |
|  | 65 | NM_004448.3，c.2313_2324dup，p.Ala775_Gly776insTyrValMetAla |
|  | 66 | NM_004448.3 c.2313_2324dup p.Ala775_Gly776insTyrValMetAla |
|  | 67 | NM_004448.3，c.2332_2340dup p.Gly778_Pro780dup |
|  | 68 | NM_004448.3 c.2313_2324dup p.Ala775_Gly776insTyrValMetAla |
|  | 69 | NM_004448.3 c.2313_2324dup p.Ala775_Gly776insTyrValMetAla |
|  | 70 | NM_004448.3 c.2313_2324dup p.Ala775_Gly776insTyrValMetAla |
|  | 71 | NM_004448.3 c.2326_2327insTGT p.Gly776delinsValCys |
|  | 72 | NM_004448.3，c.2313_2324dup，p.Ala775_Gly776insTyrValMetAla |
|  | 73 | NM_004448.3 c.2313_2324dup p.Ala775_Gly776insTyrValMetAla |
|  | 74 | NM_004448.3 c.2313_2324dup p.Ala775_Gly776insTyrValMetAla |
|  | 75 | NM_004448.3 c.2313_2324dup p.Ala775_Gly776insTyrValMetAla |
|  | 76 | NM_004448.3，c.2313_2324dup，p.Ala775_Gly776insTyrValMetAla |
|  | 77 | NM_004448.3 c.2313_2324dup p.Ala775_Gly776insTyrValMetAla |
|  | 78 | NM_004448.3，c.2326_2327insTAT，p.Gly776delinsValCys |
|  | 79 | NM_004448.3，c.2313_2324dup，p.Ala775_Gly776insTyrValMetAla |
|  | 80 | NM_004448.3，c.2313_2324dup，p.Ala775_Gly776insTyrValMetAla |
|  | 81 | NM_004448.3 c.2326_2327insTCT p.Gly776delinsValCys |
|  | 82 | NM_004448.3 c.2314_2325dup p.Tyr772_Ala775dup |
|  | 83 | NM_004448.3，c.2313_2324dup，p.Ala775_Gly776insTyrValMetAla |
|  | 84 | NM_004448.3，c.2326_2327insTGT(p.Gly776delinsValCys |
|  | 85 | NM_004448.3，c.2313_2324dup(p.A775_G776insYVMA) |
|  | 86 | NM_004448.3 ， c.2325_2329delinsATACGTGA(p.G776_V777delinsYVM） |
|  | 87 | NM_004448.3 , c.2313_2324dup(p.A775_G776insYVMA) |
|  | 88 | NM_004448.3, c.2313_2324dup(p.A775_G776insYVMA) |
|  | 89 | NM_004448.3，c.2326_2327insTCGTGATGGCTG(p.A775_G776insVVMA) |
|  | 90 | NM_004448.3, c.2325_2329delinsATACGTGA(p.G776_V777delinsYVM) |
|  | 91 | NM_004448.3， c.2313_2324dup(p.A775_G776insYVMA) |
|  | 92 | NM_004448.3，c.2313_2324dup(p.A775_G776insYVMA) |
|  | 93 | NM_004448.3, c.2314_2325dup(p.Y772_A775dup) |
|  | 94 | NM_004448.3, c.2326_2327insTGT(p.G776delinsVC) |
|  | 95 | NM_004448.3, c.2326_2327insTGT(p.G776delinsVC) |
|  | 96 | NM_004448.3, c.2313_2324dup(p.A775_G776insYVMA) |
|  | 97 | NM_004448.3, c.2326_2327insTGT(p.G776delinsVC) |
|  | 98 | NM_004448.3, c.2313_2324dup(p.Y772_A775dup) |
|  | 99 | NM_004448.3，c.2314_2325dup(p.Y772_A775dup |
|  | 100 | NM_004448.3 ，c.2326_2327insTGT (p.G776delinsVC) |
|  | 101 | NM_004448.3, c.2313_2324dup(p.Y772_A775dup) |
|  | 102 | NM_004448.3, c.2313_2324dup(p.Y772_A775dup) |
|  | 103 | NM_004448.3, c.2313_2324dup(p.Y772_A775dup) |
|  | 104 | NM_004448.3, c.2326_2327insTTT(p.G776delinsVC) |
|  | 105 | NM_004448.3, c.2313_2324dup(p.Y772_A775dup) |
|  | 106 | NM_004448.3, c.2313_2324dup(p.Y772_A775dup) |
|  | 107 | NM_004448.3, c.2313_2324dup (p.Y772_A775dup) |
|  | 108 | NM_004448.3, c.2326_2327insCTGTGGGCT(p.G776delinsAVGC) |
|  | 109 | NM_004448.3, c.2313_2324dup(p.Y772_A775dup) |
|  | 110 | NM_004448.3, c.2313_2324dup (p.Y772_A775dup) |
|  | 112 | NM_004448.3 c. 2324_2325 insATACGTGATGGC |
|  | 115 | NM_004448.3 c. 2324_2325 insATACGTGATGGC |
|  | 119 | NM_004448.3 ，c.2326_2327insTGT (p.G776delinsVC) |
|  | 120 | NM_004448.3 c.2313_2324dup p.Ala775_Gly776insTyrValMetAla |
|  | 121 | NM_004448.3 c. 2324_2325 insATACGTGATGGC |
|  | 122 | NM_004448.3 c.2313_2324dup p.Ala775_Gly776insTyrValMetAla |
|  | 123 | NM_004448.3 c.2339_2340insGGGCTCCCC |
|  | 124 | NM_004448.3 c. A775_G776insYVMA |
|  | 125 | NM_004448.3 c .2324_2325 insATACGTGATGGC |
|  | 126 | NM_004448.3 c .A775_G776insYVMA |
|  | 127 | NM_004448.3 c.2313_2324dup p.Ala775_Gly776insTyrValMetAla |
|  | 128 | NM_004448.3，c.2313_2324dup，p.Ala775_Gly776insTyrValMetAla |
|  | 129 | NM_004448.3 c.2313_2324dup p.Ala775_Gly776insTyrValMetAla |
|  | 130 | NM_004448.3 c.2313_2324dup p.Ala775_Gly776insTyrValMetAla |
|  | 136 | NM_004448.3 c.A775_G776insYVMA |
|  | 137 | NM_004448.3 , c.2313_2324dup(p.A775_G776insYVMA) |
|  | 138 | NM_004448.3, c.2313_2324dup(p.A775_G776insYVMA) |
|  | 139 | NM_004448.3，c.2313_2324dup，p.Ala775_Gly776insTyrValMetAla |
|  | 140 | NM_004448.3，c.2313_2324dup，p.Ala775_Gly776insTyrValMetAla |
|  | 141 | NM_004448.3 c.2313_2324dup p.Ala775_Gly776insTyrValMetAla |
|  | 143 | NM_004448.3, c.2313_2324dup(p.Y772_A775dup) |
|  | 144 | NM_004448.3，c.2313_2324dup，p.Ala775_Gly776insTyrValMetAla |
|  | 145 | NM_004448.3，c.2313_2324dup，p.Ala775_Gly776insTyrValMetAla |
|  | 146 | NM_004448.3, c.2314_2325dup(p.Y772_A775dup) |
|  | 147 | NM_004448.3 c.2313_2324dup p.Ala775_Gly776insTyrValMetAla |
