# Supplementary figures and images for: Anti-angiogenic therapy or immunotherapy? A real-world study of patients with advanced non-small cell lung cancer with EGFR/HER2 exon 20 insertion mutations
Source: Front Oncol. 2024 Mar 19;14:1357231. doi: 10.3389/fonc.2024.1357231 (PMC10985835; doi:10.3389/fonc.2024.1357231)

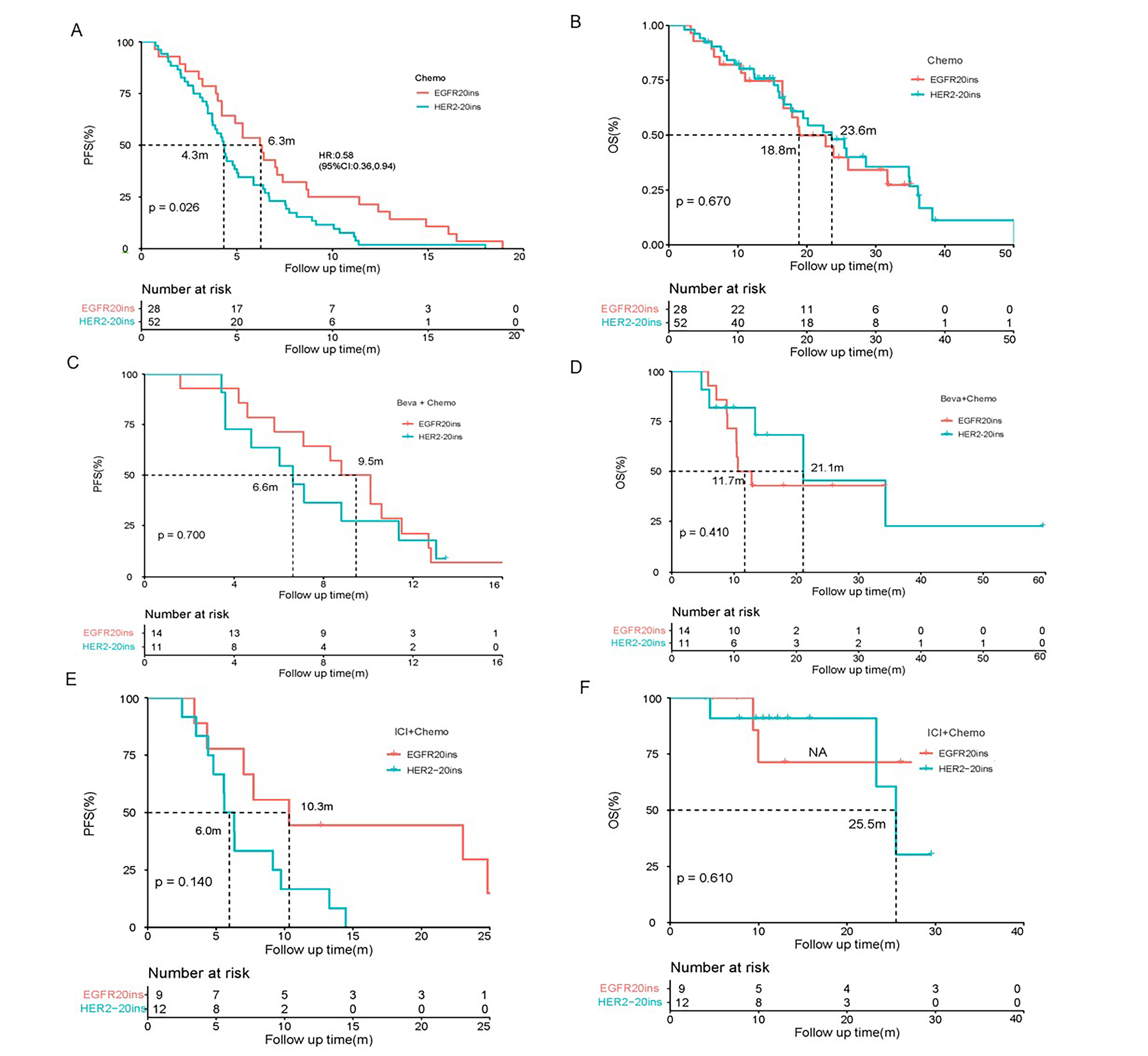

Supplement: Supplementary Figure 1 — Comparison of the efficacy of EGFR20ins and HER2-20ins on the same treatment regimen. (A, B) Comparison of PFS and OS in EGFR20ins and HER2-20ins patients receiving chemotherapy; (C, D) Comparison of PFS and OS in EGFR20ins and HER2-20ins patients receiving Beva+Chemo. (E, F) Comparison of PFS and OS in EGFR20ins and HER2-20ins patients receiving ICI+Chemo. [file Image_1.tif]

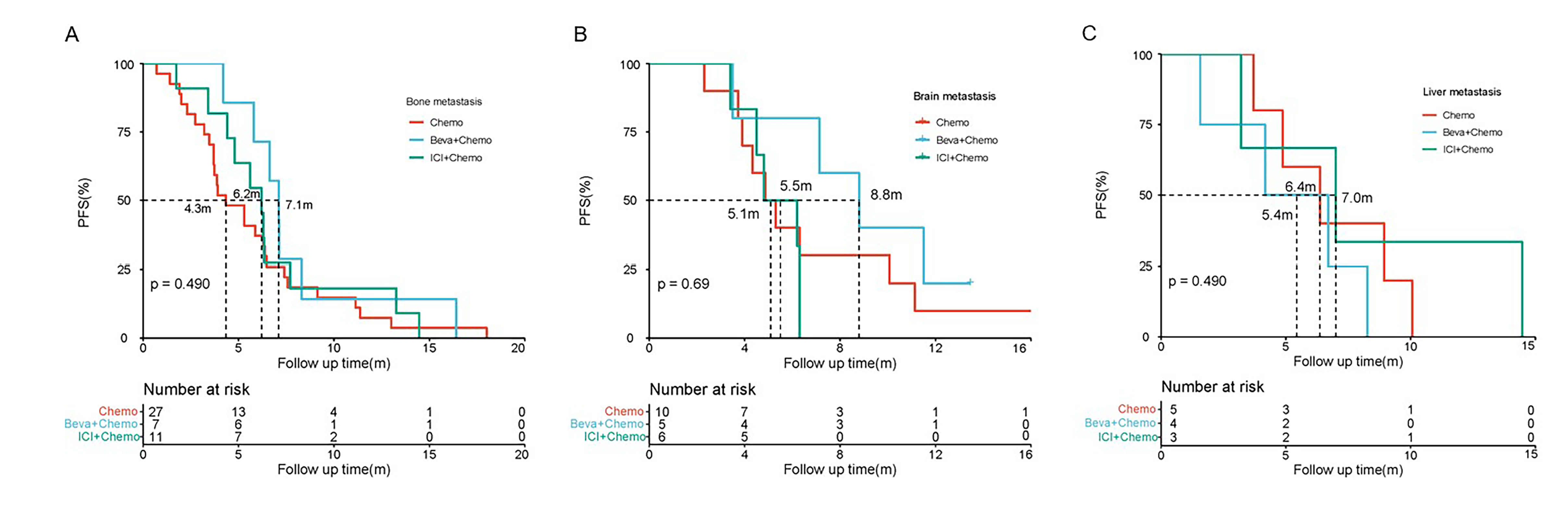

Supplement: Supplementary Figure 2 — Comparison of various treatment strategies for patients with specific metastatic sites. (A) PFS of three treatment strategies in patients with bone metastases; (B) PFS of three treatment strategies in patients with brain metastases; (C) PFS of three treatment strategies in patients with liver metastases. [file Image_2.tif]

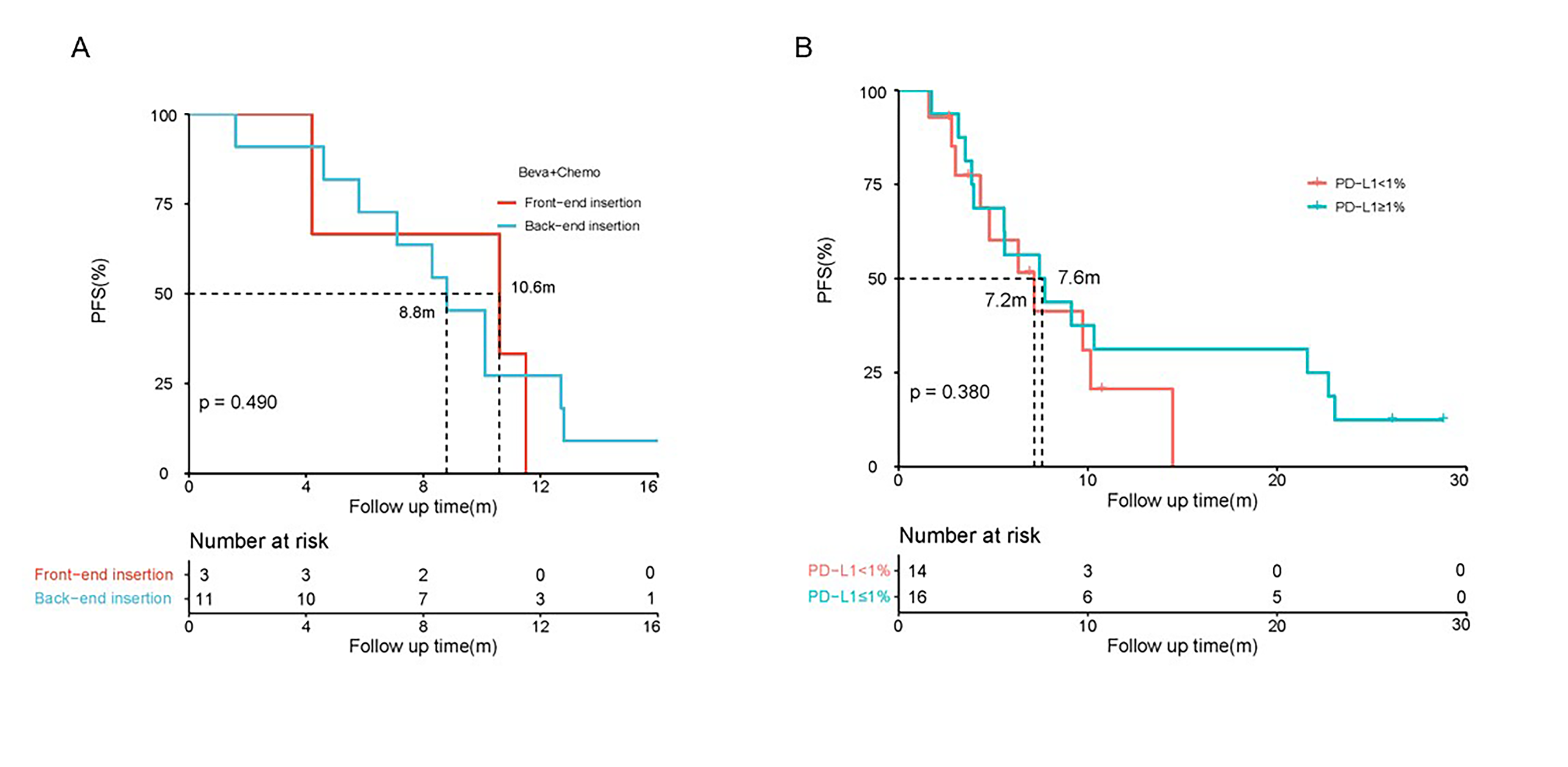

Supplement: Supplementary Figure 3 — Comparison of efficacy in different populations. (A) Comparison of PFS in patients with EGFR 20 exon front-end insertion and back-end insertion receiving Beva+Chemo. (B) Comparison of PFS between PD-L1 negative and positive patients receiving immunotherapy. [file Image_3.tif]
